# Analysis of clinical characteristics and imagological features of the aortic dissection patients with negative D-dimer results

**DOI:** 10.3389/fcvm.2023.1266919

**Published:** 2023-12-01

**Authors:** Zhixiang Zhang, Lilan Wang, Xin Su, Yuling Zhou, Kaimin Wu, Guangfeng Sun, Weimei Ou, Lihong Yu, Weifen Chen, Bin Wang

**Affiliations:** ^1^ Department of Emergency, Women and Children’s Hospital, School of Medicine, Xiamen University, Xiamen, China; ^2^ Department of Emergency, Xiamen Cardiovascular Hospital of Xiamen University, School of Medicine, Xiamen University, Xiamen, China; ^3^ Department of Emergency, Zhongshan Hospital (Xiamen), Fudan University, Xiamen, China; ^4^ The Third Clinical Medical College, Fujian Medical University, Fuzhou, China

**Keywords:** aortic dissection, D-dimer, clinical characteristics, imagological features, diagnosis

## Abstract

**Background:**

D-dimer (DD) is a vital biomarker to rule out the diagnosis of aortic dissection (AD). However, the DD level in some patients with AD is not high in clinical practice, which often leads to missed diagnosis; therefore, understanding the characteristics of patients with AD and negative DD is of great clinical value.

**Methods:**

From May 2015 to October 2020, 286 patients with AD who visited the first medical contact (FMC) within 24 h of symptom onset and were hospitalized in the Xiamen Cardiovascular Hospital of Xiamen University were enrolled in this study. Clinical characteristics and outcomes of patients were assessed.

**Results:**

Among them, 13 cases (approximately 4.5%) had negative DD results. Compared to patients with positive DD results, patients with negative DD results had significantly higher platelet counts and lower aortic dissection detection risk scores (ADD-RS). The imagological analysis showed that patients with AD and negative DD had lower extension scores and milder damage to the mesenteric artery and three branches of the aortic arch. Furthermore, the results of the multivariable analysis showed that white blood cell count (WBC) [odds ratio (OR): 1.379, *P *= 0.028], FMC (OR: 0.904, *P *= 0.028), and extension score (OR: 1.623, *P *= 0.046) were associated with negative DD result.

**Conclusions:**

Patients with AD and negative DD results had longer FMC and lower WBC. Imaging showed a smaller tear extension range and less damage to the mesenteric artery and three branches of the aortic arch. A negative DD result could not completely rule out AD even if the ADD-RS was zero.

## 1. Introduction

As shown previously, aortic dissection (AD) is one of the most life-threatening conditions caused by tears in the intimal layer of the aorta or bleeding into the aortic wall, resulting in severe aortic rupture or peripheral hypoperfusion (1). Recent epidemiological studies reported that the annual prevalence of AD is approximately 40 cases per 100,000 among people aged between 65 and 75 worldwide (2). Moreover, AD is a common fatal macrovascular disease with different clinical manifestations, which is likely to be misdiagnosed (3). Therefore, effective, rapid, and accurate diagnosis and confirmation are crucial for managing patients with suspected AD.

DD, a serum biomarker for early diagnosis of AD, can be easily detected in the emergency department (4). Previous studies demonstrated that higher serum concentrations of DD show higher sensitivity for diagnosing AD, whereas negative DD can rule out AD (5). Recent findings from different studies have confirmed that approximately 7.5% of patients with AD have negative DD results (6–8). These findings suggest that a negative DD result cannot simply rule out AD. Our study aimed to analyze the clinical characteristics and the imagological features of patients with AD and negative DD results, which may help the early diagnosis of AD in the emergency department.

## 2. Methods

### 2.1. Selection of participants

From May 2015 to October 2020, this single-center, retrospective observational study enrolled 286 consecutive patients with AD visiting the first medical contact (FMC) within 24 h of symptom onset who were admitted to Xiamen Cardiovascular Hospital of Xiamen University. AD was classified according to the Stanford standard. Type A dissection was defined as any dissection involving the ascending aorta or the arch (proximal to the left subclavian artery), and type B dissection was defined as dissection limited to the descending aorta. For patients with several episodes of AD, only the first registered episode was included in the analysis. Definite diagnosis of AD was made using thoracic and abdominal contrast-enhanced computed tomography (CT).

**Figure 1:**
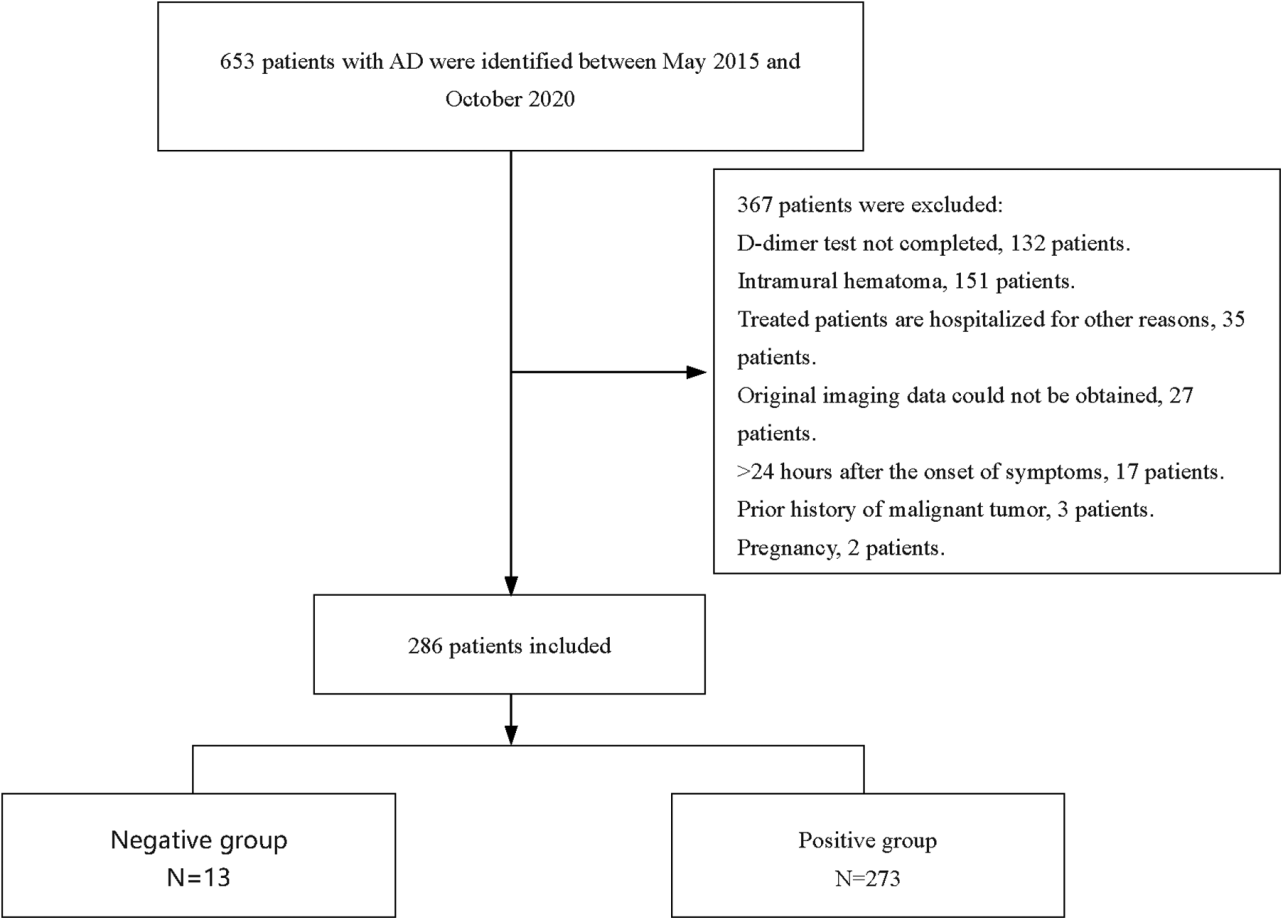
Flow chart of patient enrollment.

The exclusion criteria were as follows: (1) patients without on-admission DD result; (2) patients who were also diagnosed with intramural hematoma; (3) patients with treated AD who were hospitalized for other reasons; (4) original imaging data could not be obtained; (5) symptoms persisting for more than 24 h; (6) having a history of malignant tumors; and (7) pregnancy. A flowchart of the patients' enrollment is shown in Figure 1.

### 2.2. Study protocol

The study was approved by the Ethics Committee of Xiamen Cardiovascular Hospital of Xiamen University. The study was conducted in accordance with the revised Declaration of Helsinki. We retrospectively reviewed the medical records of patients. On admission, blood samples were obtained for routine laboratory tests.

The following factors were compared between patients with negative DD results (negative group) and those with positive DD results (positive group): age, gender, Stanford classification, FMC, past medical history, presenting symptoms, aortic dissection detection risk score (AAD-RS), laboratory results, extension score, physical and CT findings.

True lumen and false lumen diameters were measured on the same slice in the thickest part of the arterial false lumen in CT angiography. Significant involvement of aortic branches was defined as branch stenosis >50% or blood supply from false lumen. The extension score of AD in each patient was determined by considering the location of dissection in the following segments: ascending aorta, aortic arch, thoracic descending aorta, suprarenal abdominal aorta, infra-renal abdominal aorta, and iliac arteries. Scores (1–7) were calculated according to the segment involved, with the thoracic descending aorta receiving 2 scores due to its length and the remaining segments, each one receiving 1 score. ADD-RS was calculated retrospectively based on 12 clinical risk factors classified into three categories (predisposing conditions, pain features, and physical findings). The score was calculated based on the number of categories where at least one risk factor was present (9).

The results of the imaging study were interpreted by experienced radiologists and cardiologists. All patients underwent urgent CT scans for final diagnosis.

### 2.3. DD level measurement

All blood samples collected during the routine clinical evaluation were immediately sent to the laboratory for measuring DD level using the immunoturbidimetry method. Sysmex CS-5100 Automated Coagulation Analyzer from Japan and INNOVANCE reagents from Germany were used for the assay. The reference range of DD was 0–0.55 μg/mL, and patients with DD level <0.55 μg/ml were classified into the negative group, and patients with DD level ≥0.55 μg/ml were classified into the positive group.

### 2.4. Data analysis

SPSS 25.0 (IBM, Armonk, NY, USA) and GraphPad Prism 9.0 were used for statistical analysis. Continuous variables with normal distribution are described as mean ± standard deviation, and continuous variables without normal distribution are described as median and quartile. Categorical data are expressed as frequency and percentages. Independent t-test or nonparametric Mann-Whitney U test was used to compare continuous variables, whereas chi-square or Fisher exact test was applied for categorical variables. Laboratory results, CT findings, and clinical characteristics (excluding AAD-RS) with *p* < 0.05 in the univariate analysis were used in the multivariate analysis model. Odds ratio (OR) and 95% confidence interval (CI) were calculated. A *p*-value of <0.05 was considered statistically.

## 3. Results

### 3.1. Baseline characteristics of patients

**Figure 2:**
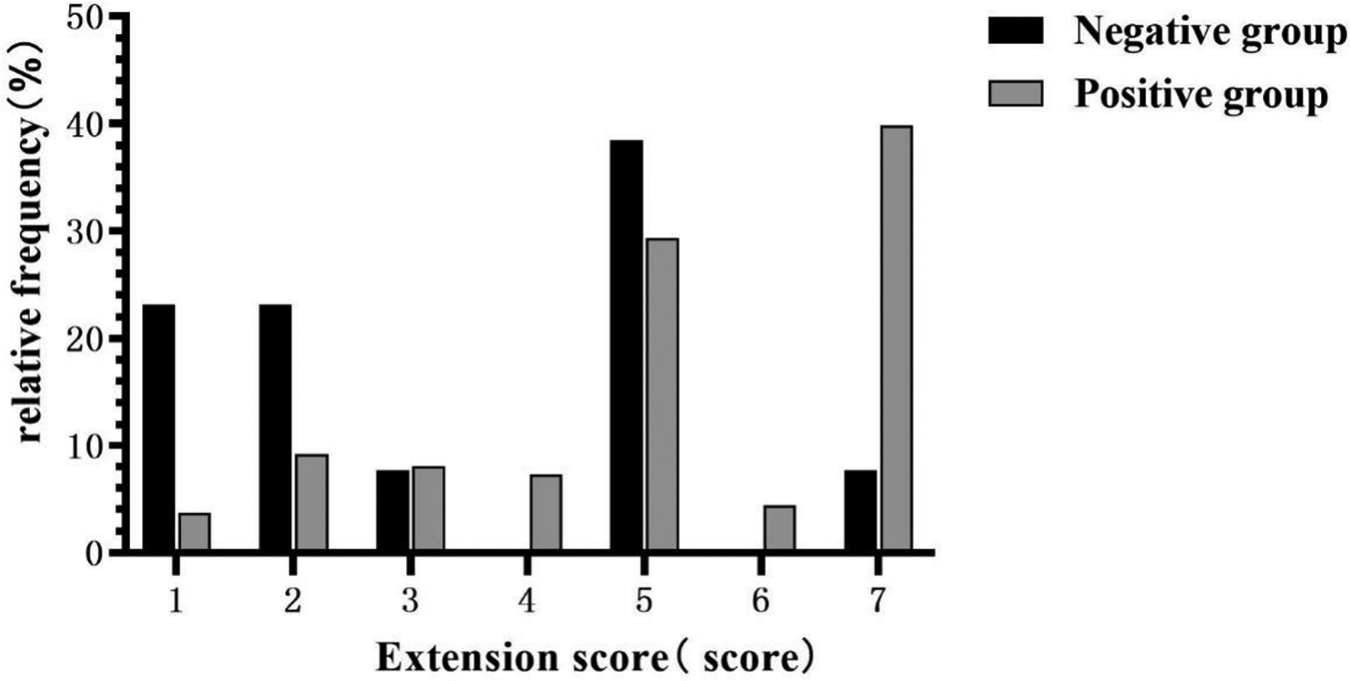
Comparison of extension scores between negative D-dimer (DD) group and positive DD group.

Based on the inclusion and exclusion criteria, 286 participants, with a median age of 53 years, were included in the final analysis (see Figure 1), most of whom were men (82.5%). The baseline characteristics of the 286 participants are shown in Table 1. In general, 13 (4.5%) and 273 (95.5%) patients showed negative and positive DD results, respectively. The median age of the negative group was 53 years, and 11 (84.6%) of them were male (as shown in Table 1). Compared with the positive group, patients in the negative group showed a significantly longer FMC period (24 vs. 6, *P *< 0.001), higher platelet count (218 vs. 167, *P *= 0.007), and relatively lower WBC (8.29 vs. 13.22, *P *< 0.001). Meanwhile, pain was milder in the negative group (46.15% vs. 97.07%, *P *< 0.001). Additionally, the ADD-RS was significantly lower in the negative group compared to the positive group (1 vs. 2, *P *< 0.001). Imaging results showed the extension score of dissection was lower in the negative group than in the positive group (3 vs. 5, *P *= 0.002). The extension scores were mainly 1–2 points in the negative group (Figure 2), while 7 points in the positive group. Moreover, the involvement of the mesenteric artery and the three branches of the aortic arch was less likely in the negative group than in the positive group.

**Table 1:** Baseline characteristics of all patients (*N* = 286).

	Negative group (*n* = 13)	Positive group (*n* = 273)	*P*-value
Gender (male)	11 (84.61%)	225 (82.42%)	>0.999
Age (year)	53 (48.00–55.00)	53 (45.00–64.00)	0.948
FMC (h)	24.00 (8.50–24.00)	6.00 (5.00–9.00)	<0.001
Temperature (°C)	36.5 (36.25–36.75)	36.5 (36.30–36.75)	0.875
Heart rate (beat/min)	80 (66.00–88.50)	79 (67.00–89.00)	0.829
Systolic blood pressure (mmHg)	156 (133.5–169.5)	148 (120.5–169.5)	0.318
Diastole blood pressure (mmHg)	90 (65.50–102.5)	81 (66.00–94.00)	0.343
Syncope or unconsciousness	0 (0%)	18 (6.59%)	>0.999
Cerebrovascular disease	1 (7.69%)	12 (4.40%)	0.461
Diabetes	1 (7.69%)	4 (1.47%)	0.209
Hypertension	12 (92.31%)	225 (82.42%)	0.704
Pain*	6 (46.15%)	265 (97.07%)	<0.001
AAD-RS	1 (1–1)	2 (1–2)	<0.001
Laboratory results			
White blood cell (10^9^/L)	8.29 (6.16–11.96)	13.22 (10.95–16.05)	<0.001
Platelet (10^9^/L)	218 (171–248.5)	167 (138–204)	0.007
Calcitoninogen (ng/ml)	0.09 (0.04–0.44)	0.15 (0.06–0.44)	0.311
Hs-cTnT (ng/L)	9.40 (7.26–23.28)	13.99 (8.19–45.90)	0.134
NT-proBNP (ng/L)	105.00 (45.75–729.57)	214.10 (95.47–729.57)	0.284
CT findings			
Extension score	3.00 (1.50–5.00)	5.00 (4.00–7.00)	0.002
True lumen diameter (cm)	1.50 (1.12–2.76)	1.43 (0.94–1.83)	0.252
False lumen diameter (cm)	2.32 (1.18–2.69)	2.36 (1.68–3.25)	0.352
Stanford type A dissection	4 (30.77%)	158 (57.88%)	0.054
False lumen with partial thrombosis	8 (61.54%)	173 (63.40%)	>0.999
Coronary artery involvement	0 (0%)	16 (5.86%)	>0.999
Three bifurcated vessels of the aortic arch involvement	2 (15.38%)	123 (45.05%)	0.035
The mesenteric artery involvement	0 (0%)	77 (28.20%)	0.023
Coeliac trunk artery involvement	3 (23.08%)	102 (37.36%)	0.385
Renal artery involvement	5 (38.46%)	161 (58.97%)	0.143
Iliac artery involvement	6 (46.15%)	170 (62.27%)	0.243

AAD-RS, the aortic dissection detection risk score; FMC, symptom onset to the first medical contact; Pain*, Contains chest pain, back pain, abdominal pain, low back pain.

### 3.2. Multivariable logistic regression analysis

WBC [odds ratio (OR): 1.379, *P *= 0.028], FMC (OR: 0.904, *P *= 0.028), and the extension score (OR: 1.623, *P *= 0.046) were associated with negative DD result (Table 2).

**Table 2:** Multivariable logistic regression models for risk factors related to negative results of D-dimer testing on the diagnosis of AD.

	OR	95% CI	*P*-value
FMC (h)	0.904	0.826–0.989	0.028
Non-pain	0.130	0.017–0.996	0.050
White blood cell (10^9^/L)	1.379	1.034–1.839	0.028
Platelet (10^9^/L)	0.988	0.974–1.002	0.087
Stanford type A dissection	0.469	0.055–4.004	0.489
Extension score	1.623	1.008–2.613	0.046
Three bifurcated vessels of the aortic arch involvement	1.098	0.112–10.733	0.936
The mesenteric artery involvement	0.000	0.000–0.000	0.997
False lumen with partial thrombosis	1.861	0.314–11.032	0.494

OR, odds ratio; 95% CI, 95% confidence interval; FMC, symptom onset to the first medical contact.

## 4. Discussion

### 4.1. Negative DD results combined with ADD-RS zero score could not completely rule out suspected aortic dissection

DD, a small fragment that can be detected after coagulation, is currently used in clinical practice for its high sensitivity; however, it has low specificity for diagnosing AD. Notably, the negative value of DD has recently been confirmed to have a high predictive power (10). Since 2007, It has been accepted that a DD value less than 0.1 mg/ml can rule out AD (11), which has been confirmed by many clinical observations from different countries (12). More recently, Yin et al. conducted a comprehensive systemic meta-analysis and found that the pooled sensitivity of DD for AD was approximately 94.5% and 69.1%, respectively, indicating that DD is the best biomarker for ruling out AD (13).

Nevertheless, recent reports have demonstrated that patients with AD can have negative DD results. Morita et al. found that among 113 consecutive patients with AD who came within 24 h of symptom onset, nine patients (8%) exhibited negative DD results (14). Additionally, approximately 45% of patients in the negative group were diagnosed with type A dissection, and 33% underwent emergency surgery due to cardiac tamponade (15), implying fatal conditions even in patients with negative DD. Therefore, we analyzed the characteristics of patients with AD and negative DD to provide a reference for the accurate and effective diagnosis of AD in patients with suspected AD. We enrolled patients admitted within 24 h of symptom onset and figured out that approximately 4.5% of patients with AD had a negative DD result. Additionally, we found that low ADD-RS was significantly associated with negative DD. Takayama et al. reported that none of the DD-negative patients had an AAD-RS score of zero (15). Stefano et al. reported that ADD-RS 0 or ≤1 combined with a negative DD can accurately rule out AD (16), whereas Ruth et al. reported two patients with acute AD who had zero AAD-RS and negative DD (17). We found 2 (15.4%) patients with a zero score in the negative group, suggesting a negative DD result can not completely rule out AD even if the ADD-RS is zero.

### 4.2. Extension score, false lumen diameter, and affected vessels in patients with negative DD result

The exposed area of the intimal layer was decided based on the length of the dissection tear and false lumen diameter. Smaller exposed area was associated with weaker activation of exogenous coagulation factors. Therefore, patients with a smaller dissection tear range and smaller false lumen diameter were more likely to have negative DD results. We divided the aorta into several segments in a relatively average way, and the extension score was calculated based on the number of these segments, which could indirectly indicate the length of the dissection tear. Our imagological analysis showed that there were lower extension scores, smaller false lumen diameters, and milder involvement of the mesenteric artery and branches of the aortic arch in the negative group than in the positive group.

Damages to peripheral organs supplied by the three branches of the aortic arch and the mesenteric artery were associated with large dissection areas in patients with AD. Thus, the involvement of these vessels caused a large area of hypoperfusion, damaging vascular endothelial cells and activating endogenous coagulatory pathways (18). Thus, severe dissection can present with increased serum concentrations of DD. Consistently, we found a lower extension score in AD patients with negative DD results, suggesting milder organ ischemia. Additionally, our imagological analysis might provide a possible explanation for Chai X et al.' outcome that increased DD concentrations can predict a higher risk of in-hospital mortality in patients with AD (19).

### 4.3. Inflammatory response in patients with AD and negative DD results

During the development of AD, the inflammatory response is involved in several pathological processes in the affected artery, including medial degradation of the aortic artery and arterial wall remodeling, which subsequently weaken the aortic wall and increase mortality (20). On the other hand, the imbalance between pro-inflammatory and anti-inflammatory signals can contribute to AD (21). Takayama et al. demonstrated that WBC significantly increases in patients with AD owing to the inflammatory response in the acute phase reaction (15). Recently, some studies have shown a wide range of interactions between inflammatory response systems and vascular systems. The inflammatory response not only stimulates coagulation but also accelerates the progression of coagulation (22, 23). Previous clinical trials on patients with AD demonstrated that increased concentrations of DD can reflect the severity of systemic inflammatory response (24). Besides, it was shown that patients with AD and increased WBC possess higher levels of DD (25). Similarly, another clinical trial reported that WBC is increased in patients with positive DD (19). We have shown that patients with negative DD results have a lower WBC and a higher platelet count compared with those with positive DD, possibly due to lower tear extension scores in the negative group. Because of the smaller tear extension score, the exposure area of the intima of the artery is relatively smaller, and the elevating count of WBC caused by both the acute phase reaction as well as the underlying inflammatory process is smaller (26), resulting in a lower WBC count in the negative group. Additionally, as the exposed area of the intimal layer was smaller in the negative group, coagulation and platelet aggregation were less likely in this group, resulting in a higher platelet count.

Multivariable logistic regression also showed that a low WBC is associated with a negative DD result. These findings also shed light on the underlying processes of the inflammatory response is inclined to become the targets for treating AD in the future.

### 4.4. Painless AD in the negative DD group

Typically, AD presents with acute or severe chest, back, and tearing abdominal pain. It has also been suggested that AD can be rarely painless (27). Imamura et al. demonstrated that AD can be painless due to neurologic deficit, syncope, or disturbance of consciousness (28). Besides, slow or gradual dissection with less wall stretching can be painless. Though there are several potential explanations for the absence of pain, none are convincing (25). Since the negative group presented a smaller extension range with less wall stretching, asymptomatic AD is expected to be more common in this group.

### 4.5. Others

We found that among patients with negative DD results, the FMC of nine patients (approximately 69.2%) was between 20 and 24 h, which was slightly longer than that in patients with positive DD results. In addition, the results of the multivariable analysis showed that FMC (OR: 0.904, *P *= 0.028) was associated with negative DD results. Eggebrecht et al. reported that FMC is inversely associated with the serum concentrations of DD in patients with AD (29). However, they did not provide a reasonable explanation for the underlying mechanism. Thus, it is still needed to conduct in-depth investigations.

Cai Y et al. showed a statistically significant association between relatively low blood pressure and negative DD results (19), but our study did not find a similar association. Murai M et al. reported that age is an independent risk factor for positive DD, and none of the patients with negative DD were older than 70 years in their study (14). However, in our study, two patients with negative DD were 73 years old and 81 years old, and age did not significantly differ between the positive and negative groups.

## 5. Conclusions

Patients with AD and negative DD had longer FMC and slighter chest pain. Imaging showed a smaller tear extension range and less involvement of the mesenteric artery and three branches of the aortic arch. In clinical practice, physicians should be aware that a negative DD result cannot completely rule out AD even if the ADD-RS is zero. Therefore, imaging should be conducted as early as possible for patients with suspected AD.

## 6. Study limitations

There are some limitations to this study. First, it was a single-center retrospective study with a relatively small number of AD patients in the negative group. Second, participants with both Stanford type A and type B AD were included. Given the small number of participants, we did not conduct an independent analysis on patients with type A or B dissection. Nevertheless, our findings are important since the molecular mechanisms through which D-dimer is produced are similar between Stanford type A and type B aortic dissection. Finally, as we focused on Chinese patients, similar studies on other nationalities are needed.

## Data Availability

The original contributions presented in the study are included in the article/Supplementary Material, further inquiries can be directed to the corresponding author.
